# The Role of Proton Pump Inhibitors in the Realm of Idiopathic Pulmonary Fibrosis and its Associated Comorbidities: A Systematic Review

**DOI:** 10.7759/cureus.55980

**Published:** 2024-03-11

**Authors:** Sadaf Iftikhar, Sarah F Alhaddad, Christian N Paulsingh, Muhammad Faisal Riaz, Gourav Garg, Lotanna Umeano, Pousette Hamid

**Affiliations:** 1 Internal Medicine, California Institute of Behavioral Neurosciences & Psychology, Fairfield, USA; 2 Pediatrics, California Institute of Behavioral Neurosciences & Psychology, Fairfield, USA; 3 Pathology, St. George's University School of Medicine, St. Georges, GRD; 4 Internal Medicine, Rawalpindi Medical University, Rawalpindi, PAK; 5 Orthopaedics, Kings Mill Hospital, Sutton in Ashfield, GBR; 6 Neurology, California Institute of Behavioral Neurosciences & Psychology, Fairfield, USA

**Keywords:** idiopathic pulmonary fibrosis and its comorbidities, antacid, pirfenidone, omeprazole, proton-pump inhibitors (ppi), gastroesophageal reflux disease (gerd), idiopathic pulmonary fibrosis

## Abstract

As the global incidence of idiopathic pulmonary fibrosis (IPF) is on the rise, there is a need for better diagnostic criteria, better treatment options, early and appropriate diagnosis, adequate care, and a multidisciplinary approach to the management of patients. This systematic review explores the role of proton pump inhibitors (PPIs) in IPF and answers the question, “Does proton pump inhibitor improve only the prognosis of gastroesophageal associated idiopathic pulmonary fibrosis or for other types of idiopathic pulmonary fibrosis too?” We used PubMed (PMC) and Google Scholar for data collection for this systematic review and followed the Preferred Reporting Items for Systematic Reviews and Meta-Analyses (PRISMA) guidelines for conducting this review. After in-depth literature screening and quality appraisal, 12 articles were selected for this systematic review. On the one hand, the efficacy of PPI therapy is supported by research such as the CAPACITY and ASCEND trials, a pilot randomized control trial (RCT) investigating the role of omeprazole in IPF and a bidirectional two-sample Mendelian randomization (MR) study, respectively. On the other hand, a systematic review and meta-analysis on antacid and antireflux surgery in IPF negate these results and show no statistical significance. Questions regarding the efficacy of PPI therapy must be dealt with in an adequately powered multicenter and double-blinded randomized control trial. The anti-inflammatory properties of antacids can serve as the cornerstone for future trials. In the following systematic review, antacid, antireflux therapy, omeprazole, and proton pump therapy are synonymous with stomach acid suppression therapy.

## Introduction and background

"Life" is a word that means "we are here for a short time''. When someone is diagnosed with idiopathic pulmonary fibrosis (IPF), their life expectancy falls even more because in many cases, they come to know about it late. This is because symptoms can include having no symptoms, mild cough, and shortness of breath when exerting oneself, as well as severe cough and shortness of breath while resting. Moreover, there aren't many treatment options available. People with IPF usually live for three to five years. Nintedanib and pirfenidone are accepted at present as treatments, but they are nearly impossible to get, are expensive [1], and can have significant side effects on the liver. So, it is vital to have drugs that work, are easy to get, have few side effects, and are relatively less expensive.

Gastroesophageal reflux disease (GERD) is more common in people with IPF than in the general population [2,3]. Therefore, the positive effects of antacids were looked into. One study suggests that antacid use over time leads to a decrease in radiological findings and improved survival [4]. Another study revealed that patients taking antacids have lower levels of reduced forced vital capacity and fewer acute exacerbation episodes [5]. We looked at people with IPF who were given pirfenidone in three studies: CAPACITY (PIPF-004/PIPF-006), ASCEND (PIPF-016), and a post hoc analysis where patients with IPF were treated with antacid therapy (AAT) plus pirfenidone had similar outcomes as those who were treated with pirfenidone alone. This shows that further prospective studies are needed to determine how well AAT works with or without antifibrotic drugs (nintedanib and pirfenidone) as a treatment for IPF [6]. Regarding other treatment options, a lung transplant may look like the most effective answer. However, the disadvantages of this treatment include advanced age, multiple health problems, unpredictable disease development, life-threatening acute exacerbations, and shortage of organs [7]. A systematic review of two studies found that antacids did not have a statistically significant effect on the progression of the disease (RR, 0.88; 95% CI, 0.76-1.03) [8]. Another study that was not incorporated in the meta-analysis found that antacids either had no effect or increased disease progression, depending on how it was defined. In some people, taking antacids made nonacid reflux worse and didn't improve gastroesophageal reflux (GER). Surgery to stop acid reflux limited acid exposure but did not improve GER symptoms. Antireflux surgery and antacid drugs are both conditionally advised for IPF [9].

Through this systematic review, we aim to discover the efficacy of proton pump inhibitors (PPIs) as a treatment option as well as their pros and cons. We will find out what role PPIs play as an alternative treatment option. We discover how the anti-inflammatory properties of PPIs are beneficial and how different etiologies influence the progression of IPF. Time will tell if they will stay as a treatment choice or be replaced by better and newer therapies.

## Review

Methods

We conducted our systematic review using the Preferred Reporting Items for Systematic Reviews and Meta-Analyses (PRISMA) guidelines [10].

 Search Sources and Search Strategy

We started our research on August 14, 2023, using online libraries as our database. We searched PubMed, Medline, and Google Scholar for our data collection. Our detailed search strategy, keywords, and Medical Subject headings (MeSH) are outlined in Table 1. 

**Table 1:** Search strategy. PPI: proton pump inhibitors; GERD: gastroesophageal reflux disease.

Database	Search Strategy	Number of records before applying filters	Filters applied	Results
PubMed/Medline	Mesh strategy ("Idiopathic Pulmonary Fibrosis"[MeSH Terms] OR "Cryptogenic Fibrosing Alveolitis" OR "Familial Idiopathic Pulmonary Fibrosis" OR "Fibrocystic Pulmonary Dysplasia" OR "Fibrosing Alveolitis, Cryptogenic" OR "Idiopathic Fibrosing Alveolitis, Chronic Form" OR "Idiopathic Pulmonary Fibrosis" OR "Idiopathic Pulmonary Fibrosis, Familial" OR "Interstitial Pneumonitis, Usual" OR "Pulmonary Fibrosis, Idiopathic" OR "Usual Interstitial Pneumonia") AND ("Gastroesophageal Reflux"[MeSH Terms] OR "Esophageal Reflux" OR "GERD" OR "Gastric Acid Reflux" OR "Gastric Acid Reflux Disease" OR "Gastroesophageal Reflux" OR "Gastroesophageal Reflux Disease" OR "Gastro-oesophageal Reflux" OR "Gastroesophageal Reflux" OR "Gastroesophageal Reflux Disease" OR "Reflux, Gastroesophageal") AND ("Proton Pump Inhibitors"[MeSH Terms] OR "Proton Pump Inhibitor" OR "Proton Pump Inhibitors") AND ("Humans"[MeSH Terms] OR "Homo sapiens" OR "Human" OR "Humans" OR "Man (Taxonomy)" OR "Man, Modern")	103	2013-2023, Free full text, English, clinical trial, meta-analysis, review, systematic review, RCT	9
Google Scholar	Does PPI improve the prognosis of GERD-associated idiopathic pulmonary fibrosis or for other IPF etiologies?	1110	2019-2023, review article	450

Inclusion Criteria

We selected peer-reviewed articles and studies that were published in the English language. We included only human studies in systematic reviews, traditional reviews, meta-analyses, and randomized trials. All data was collected ethically and legally.

Exclusion Criteria

We excluded grey literature and animal studies.

*Data Extraction*

Data extraction was done individually from the final articles after quality assessment.

Results

Search Results

**Figure 1:**
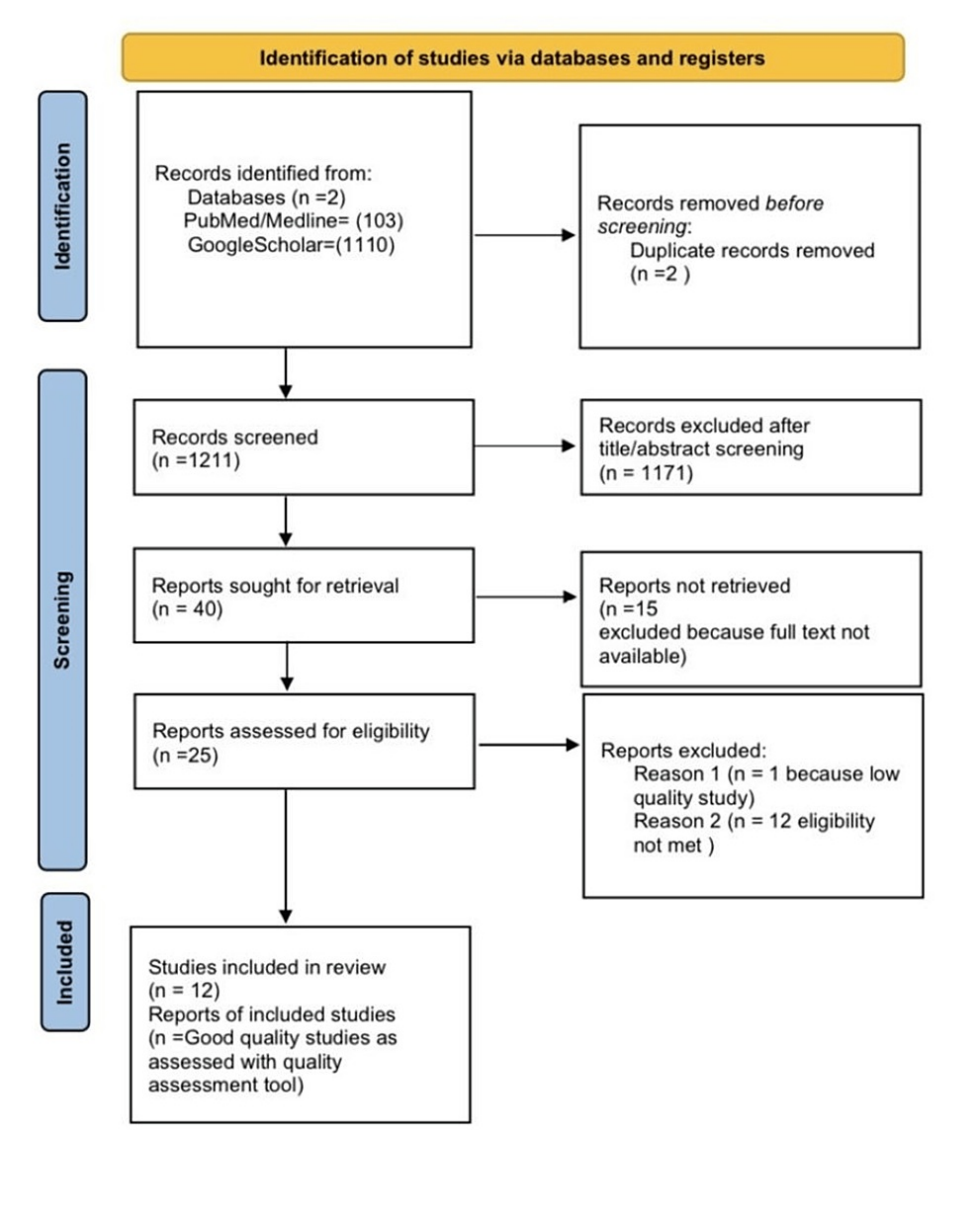
PRISMA flow diagram PRISMA: Preferred Reporting Items for Systematic Reviews and Meta-Analyses

Our extensive literature search yielded 1213 articles through PubMed and Google Scholar. We then filtered the articles and removed two duplicates, leaving 1211 articles. Two individual reviewers independently eliminated 1171 articles for being irrelevant according to title and abstract. Ultimately, 40 articles were selected for critical appraisal. We found 15 abstracts without full text and excluded them. We both individually assessed 25 articles for eligibility, and after reviewing the full articles, we excluded 12 because they were irrelevant to our study. We used various quality assessment tools on the remaining 13 articles. We excluded one low-quality article and included 12 articles for data extraction. Figure 1* *shows a detailed PRISMA flow diagram.

Critical Appraisal

We used AMSTAR (A MeaSurement Tool to Assess systematic Reviews) for the meta-analysis [11], the Cochrane risk-of-bias tool for randomized control trials [12], and the SANRA (Scale for the Assessment of Narrative Review Articles)[13], and the Newcastle-Ottawa Scale for retrospective cohort study [14]. Studies that scored above 60% were included (Table 2).

**Table 2:** Critical appraisal of included studies. SANRA: scale for the quality assessment of narrative review articles; AMSTAR: A Measurement Tool to Assess Systematic Review

AUTHOR	TITLE	STUDY TYPE	QUALITY ASSESSMENT TOOL	SCORE
Glassberg et al. [1]	Overview of Idiopathic Pulmonary Fibrosis, Evidence-Based Guidelines and Recent Developments in the Treatment Landscape	Narrative review	SANRA checklist	60%
Kreuter et al. [6]	Antacid Therapy and Disease Progression in Patients With Idiopathic Pulmonary Fibrosis Who Received Pirfenidone	Randomized control trial	Cochrane risk-of-bias tool	70%
Khor et al. [8]	Antacid Medication and Antireflux Surgery in Patients With Idiopathic Pulmonary Fibrosis: A Systematic Review and Meta-Analysis	Meta-analysis	AMSTAR	70%
Ghebre et al. [15]	Proton Pump Inhibitors in IPF: A Call for Clinical Trials	Narrative review	SANRA checklist	70%
Ghebre et al. [16]	Idiopathic Pulmonary Fibrosis: Novel Concepts of Proton Pump Inhibitors as Antifibrotic Drugs	Narrative review	SANRA checklist	70%
Ruaro et al. [17]	Gastroesophageal Reflux Disease in Idiopathic Pulmonary Fibrosis: Viewer or Actor? To Treat or Not to Treat?	Narrative review	SANRA checklist	70%
Dutta et al. [18]	Randomized, Double-Blind, Placebo-Controlled Pilot Trial of Omeprazole in IPF	Randomized control trial	Cochrane risk-of-bias tool	60%
Lee et al. [19]	Protective Effect of Proton Pump Inhibitors for Survival in Patients With Gastroesophageal Reflux Disease and Idiopathic Pulmonary Fibrosis	Retrospective cohort study	Newcastle-Ottawa Scale	Fair quality study (5 stars)
Reynolds et al. [20]	The Causal Relationship Between Gastro-Oesophageal Reflux Disease and Idiopathic Pulmonary Fibrosis: A Bidirectional Two-Sample Mendelian Randomization (MR) Study	Narrative review	SANRA checklist	75%
Tran et al. [21]	The Effect of Antacid Therapy on Survival in Idiopathic Pulmonary Fibrosis: a Methodological Review of Observational Studies	Narrative review	SANRA checklist	70%
Luppi et al. [22]	Idiopathic Pulmonary Fibrosis Beyond the Lung: Understanding Disease Mechanisms to Improve Diagnosis and Management	Narrative review	SANRA checklist	60%
Kreuter et al. [23]	Impact of Comorbidities on Mortality in Patients With Idiopathic Pulmonary Fibrosis	Retrospective cohort study	Newcastle-Ottawa Scale	Good quality study (6 stars)

Study Characteristics

After the screening and quality check, 12 articles were finally included for systematic review. The study characteristics are shown in detail in Table 3.

**Table 3:** Characteristics of included studies. JRS, Japanese Respiratory Society; ATS, American Thoracic Society; ERS, European Respiratory Society; ALAT, Latin American Thoracic Association; IPF, idiopathic pulmonary fibrosis; GERD, gastroesophageal reflux disease; MR, Mendelian randomization; LARS, laparoscopic antireflux surgery; PPIs, proton pump inhibitors; GER, gastroesophageal reflux; MII-pH, multichannel intraluminal impedance-pH testing; RCT, randomized control trial; FVC, forced vital capacity; AAT, antacid therapy

AUTHOR	JOURNAL NAME	YEAR	COUNTRY	RESULTS
Glassberg et al. [1]	American Journal of Managed Care	2019	-	The Japanese Respiratory Society (JRS) 2018 Guideline recommendations and those from the American Thoracic Society (ATS), European Respiratory Society (ERS), JRS, and Latin American Thoracic Association (ALAT) of 2015 guidelines only currently support nintedanib, pirfenidone, and antacid drugs.
Kreuter et al. [6]	Respiration	2017	Germany	A post hoc analysis of the trial (CAPACITY PIPF-004/PIPF-006) and ASCEND PIPF-016 was conducted. A relative FVC reduction of greater than 10% favored AAT (15 vs. 22%, p=0.03). AAT and pirfenidone showed similar effects, indicating the need for further trials investigating PPIs with or without pirfenidone.
Khor et al. [8]	Annals of the American Thoracic Society	2022	United States	When two trials were combined, antacid treatment did not significantly affect disease progression, defined as a 10% or more loss in FVC, more than 50-m decline in 6-minute walking distance, or mortality (RR, 0.88; 95% CI, 0.76–1.03). Antacids and antireflux surgery may not enhance respiratory outcomes in IPF patients, most of whom have typical GER.
Ghebre et al. [15]	Frontiers	2018	-	Based on retrospective data, antacids are recommended for IPF treatment irrespective of the diagnosis of GERD. Because the data is retrospective, the value is low. Antacid go above and beyond acid suppression and have a potential role in treating IPF. LARS is a good treatment option.
Ghebre et al. [16]	American Journal of Respiratory and Critical Care Medicine	2016	United States	This article shows that PPIs may treat IPF but not stomach reflux or microaspiration. PPIs' advantages while treating IPF may be due to the down-regulation of fibroinflammatory molecules, up-regulation of cytoprotective processes, reduced fibroblast proliferation, and stomach acidity control. New studies might focus on extraintestinal PPIs. Controlled clinical studies involving patients with typical and atypical GER, symptomatic and asymptomatic gastroesophageal reflux disease GERD, acidic and nonacidic reflux, and symmetrical and asymmetrical illness are required to prospectively explore the anecdotal and perhaps reflux-independent efficacy of PPIs in IPF.
Ruaro et al. [17]	Multidisciplinary Digital Publishing Institute	2022	Italy	This article supports the gastroesophageal reflux disease GERD-IPF theory. A high-resolution manometry and MII-PH should investigate the patients. The benefits of PPI therapy, which are evaluated in the latest guidelines, need to be investigated further via prospective and randomized control trials. LARS seems to be a promising treatment option for these patients.
Dutta et al. [18]	Thorax	2019	UK Newcastle	To our knowledge, this is the first pilot randomized control trial (RCT) of Omeprazole for IPF cough. The pilot experiment did not have enough power to identify statistically significant changes. However, the Omeprazole group had a lower cough rate on average. Omeprazole reduced objective cough frequency by a clinically significant amount, equivalent to other chronic cough therapies.
Lee et al. [19]	Journal of Neurogastroenterolgy and Motility	2016	South Korea	Long-term use of PPIs was shown to be linked to a decrease in death rate due to IPF in comparison to earlier research. While there was no significant link between GERD and the use of PPIs for two or three months, using PPIs for more than four months was associated with a decreased mortality due to IPF compared to using them for less than four months.
Reynolds et al. [20]	European Respiratory Journal	2023	United States, London, Spain, Italy, Australia	A bidirectional two-sample MR was conducted to evaluate the causative relationship between GERD and IPF risk, utilizing genetic data from the biggest genome-wide association meta-analyses of GERD (78 707 cases and 288 734 controls) and IPF (4125 cases and 20 464 controls). Results revealed GERD increased IPF risk by 1.6 (95% CI 1.04–2.49; p=0.032).
Tran et al. [21]	European Respiratory Journal	2018	-	The observed positive role of PPIs on the death of patients with IPF is due to lead time bias in observational studies. So, the role of antiacid treatment in IPF has not yet been approved and requires to be reinvestigated by further research.
Luppi et al. [22]	Respiratory Research	2021		Due to aging, genetics, and various environmental risk factors like cigarette smoke, IPF is most likely accompanied by comorbidities that affect the clinical picture and survival. Comorbidities negatively affect many IPF outcome factors. So, diagnosing and treating comorbidities may improve IPF patients' survival.
Kreuter et al. [23]	PLoS One	2016	Germany	In a multivariate Cox proportional hazard analysis, additional heart illnesses and lung cancer were significant predictors of mortality, although GERD and diastolic dysfunction improved survival. No survival benefit was seen with comorbidity-related therapy. PPIs at baseline did not improve survival (p = 0.718).

 Discussion

Studies Highlighting the Positive Impact of PPI in IPF

Multiple studies reveal the positive effect of PPIs on IPF. Various international guidelines only recommend pirfenidone, nintedanib, and proton pump inhibitors as medical treatment options for IPF [1]. The CAPACITY and ASCEND trials assessed a database of 624 patients to reveal positive results regarding IPF-related mortality and all-cause mortality [15,24]. A separate division of CAPACITY and ASCEND comparing pirfenidone with pirfenidone plus antacid reveals a statistically lower forced vital capacity (FVC) by 10% in those taking pirfenidone plus antacid [6,15]. PPI use in IPF may be advantageous due to the potential to reduce fibroinflammatory chemicals, increase cytoprotective mechanisms, limit fibroblast growth, and reduce stomach acidity [16]. A double-blinded, randomized, placebo-controlled pilot trial of the PPI omeprazole on 40 participants (23 on omeprazole and 22 on placebo) was conducted and the primary outcome was cough frequency. Results reveal baseline cough frequency in four hours was 8.2 and 9.1 in the omeprazole and placebo groups. Study results reveal cough frequency to be 4.6 per hour in the omeprazole group and 8.3 per hour in the placebo group. Cough frequency was 39% lower in the omeprazole group compared to placebo*.*

*Studies Highlighting the Negative Impact of PPIs in* *IPF*

The negative impact of PPIs in IPF is highlighted in a meta-analysis which reveals that post hoc analysis of two observational studies reveal that antacids don't cause a much decrease in FVC; six-minute walking duration and antireflux surgery revealed similar results [8]. Two-sample bidirectional Mendelian randomization (MR) reveals GERD is the underlying cause of IPF and not vice versa [20]. The positive impact of PPIs in observational studies is blurred by lead time bias [21]. The presence of GERD is linked with survival, and treatment does not play much role in its survival [23].

Protective Role of PPIs

PPIs promote the formation and activity of antioxidant stress proteins like heme oxygenase-1 and superoxide dismutase, scavenge reactive oxygen species, and prevent glutathione depletion, according to several molecular and cell biological studies [25]. PPIs also decrease inflammatory chemicals in nongastric cells, such as vascular endothelium and tracheal epithelial cells. PPI-regulated molecules include integrin superfamily members integrin αMβ2 (CD11b) and CD18 integrin β2 (CD18), adhesion molecules intercellular adhesion molecule (ICAM-1), vascular cell adhesion molecule (VCAM-1), and proinflammatory cytokines tumor necrotic factor (TNF-α), interleukin 1 beta (IL-1β), interleukin (IL-6) and interleukin (IL-8) [26,27]. In addition to this, PPIs directly decrease inflammatory cell motility and contact with vascular endothelial cells [26]. Lee et al., in a study conducted at Seoul National University in the Republic of Korea, examined data from 786 IPF patients in their interstitial lung disease database and found that proton pump inhibitor use was progressively associated with lower IPF-related mortality, with the benefit of drug lasting longer than two or three months resulting in longer survival times. Surprisingly, their univariate and multivariate Cox regression analysis revealed that decreased IPF was related to PPI use, not gastroesophageal reflux disease GERD diagnosis. This highlights that PPI plays a role in treating IPF irrespective of etiology [19].

The Mystery Surrounding Gastroesophageal Reflux Disease in IPF

According to retrospective research, IPF patients with GERD had a much greater median survival time than IPF patients without GERD [4]. Although the exact reasoning behind this conclusion is unknown, it might have to do with a lead time bias effect, where individuals who get a diagnosis of GERD-related pulmonary symptoms earlier in life tend to live longer than those who receive a diagnosis later. Another topic of discussion is the possible effect of antacid medication, namely proton pump inhibitors, on the course of IPF. According to a retrospective review, people with IPF who take antacid treatment tend to live longer [4]. Only 25-65% of IPF patients with a confirmed GERD diagnosis have the classic GERD symptoms, such as heartburn; however, an IPF patient's lack of signs does not rule out a GERD diagnosis [4]. Even though oesophageal manometry and 24-hour pH monitoring may help diagnose GERD, the optimal diagnostic flowchart for IPF patients to diagnose GERD is unclear and should be customized. GERD treatment is often initiated based on GER symptoms primarily because of this reason [28]. 

IPF and Its Various Comorbidities

Diabetes: The association of diabetes mellitus with IPF is based on the idea that diabetes causes IPF. Our understanding of a stronger link between insulin and IPF than with oral hypoglycemics resonates with the idea that Type 1 and Type 2 diabetes are two different types of exposures that have various effects on the development of IPF or that the early onset of the exposure is important. Therefore, doctors should be aware that people who have been diagnosed with IPF may additionally have diabetes mellitus [29].

Cardiovascular disease: A high-resolution chest CT scan is required for all patients with suspected IPF to confirm the diagnosis. Because of this, it can also be used to check for coronary artery disease (CAD) in high-risk groups. Moderate to severe calcifications in the coronary arteries can be found on high-resolution CT scans (HRCT), which makes the diagnosis of CAD very accurate [30]. If moderate to severe coronary calcifications are seen on HRCT, this radiological finding should make doctors think about sending IPF patients to a cardiologist. The co-occurrence of IPF with coronary artery disease is very common. This may be because of a genetic link between the two conditions. This link can be seen in the immune system's response to the alpha one chain of collagen type V and its relationship with human leukocyte antigen (HLA-D15) alleles [31]. However, a history of smoking and a higher rate of diabetes mellitus in these patients may also play a part.

Pulmonary hypertension: Pulmonary hypertension is defined by a resting mean pulmonary artery pressure (mPAP) equal to or above 20 mmHg, as determined by right cardiac catheterization. The average mean pulmonary arterial pressure (mPAP) is 14 mmHg with a standard variation of 3 mmHg. Pulmonary hypertension (PH) is a common comorbidity in patients with IPF, especially in later stages, and is considered an indicator of an unfavorable prognosis [32,33]. Subsequently, a thorough investigation conducted on a cohort of over 6500 patients revealed that even a small presence of pulmonary hypertension in individuals with IPF significantly elevates the likelihood of mortality [34]. Many studies indicate that precapillary pulmonary hypertension (PH) in IPF may affect about 8-15% of patients at the first evaluation [35,36]. This tends to increase in IPF patients with advanced and end-stage illness when PH becomes a dominant characteristic, affecting more than 50-60% of individuals [37,38,39].

Lung cancer: Small sample numbers restricted the studies reporting mortality and survival among IPF patients with lung cancer; however, those with concurrent lung cancer had a worse survival rate than individuals with either idiopathic interstitial pneumonia [40] or IPF alone [41]. Due to their lowered tolerance to cancer treatments, IPF patients with lung cancer are not often excellent candidates for routine therapy. As IPF patients often have a poor prognosis, management of lung cancer in this population should be done on a single-patient basis, weighing the possibility of therapeutic comorbidities against the chance of treatment [42]. After thoracic surgery, including lung cancer resection, patients with pulmonary fibrosis have a significantly increased chance of dying [43]. This increased risk is often secondary acute exacerbation of IPF (AE-IPF), which is present in 10% of IPF patients after thoracic surgery, with a high short-term mortality (approximately 50%) [44]. The exact mechanism by which surgical procedures may cause an AE is presently not known. AE-IPF was also discovered in patients treated with chemotherapy for lung cancer or following radiotherapy [45,46].

Depression and anxiety: Anxiety and depression are common (31% and 23%, respectively) [47]. Hence, all IPF patients should be examined for these disorders [28]. Due to underestimates and underdiagnosis, only 25% of anxiety and depression patients were given pharmacologic treatment, indicating that many remain untreated despite a high incidence [48]. Cognitive behavioral therapy and antidepressants may enhance the quality of life in IPF patients, although their efficacy is unknown [49]. All IPF patients with depression and functional impairment should undergo pulmonary rehabilitation since it improves depressed symptoms over time [50]. A palliative care intervention reduced anxiety and sadness in advanced fibrotic lung disease patients, demonstrating the relevance of supportive care [51].

Role of Genetics in the Association of Gastroesophageal Reflux Diseases With IPF

A recent meta-analysis of 18 case-control studies, including 326 IPF patients and 9368 controls, showed that chances of developing IPF were 2.94 times higher in those with GERD (95% CI 1.95-4.42), but there was much variation across the studies (I^2^ =86%; heterogeneity; p< 0.00001) [52]. The author did describe that confounding due to smoking caused by two factors; firstly, the initial results became insignificant after a post hoc meta-regression analysis that accounted for smoking (odds ratio [OR] 0.66, 95% confidence interval [CI] (037-1.27). Secondly, studies with a large number of patients and a smaller number of controls who smoked had bigger effect sizes. A strong link was present between the ratio of smokers, including ex-smokers, in cases of IPF compared to the control logarithmic odd ratio between GERD and IPF.

Using the MR technique, our study eliminated smoking and any other potential factor that could alter the observational studies. This confidently allows us to establish cause and effect relationship between GERD and IPF. Since a genetic link to GERD is inherent from birth, with MR, we evaluate an individual's lifetime tendency to develop GERD at the risk of IPF. Our results are not affected by any dietary or pharmacological therapy for GERD [20].

Limitations

This systematic review has found limited data pertaining to the effects of antacids on other etiologies of IPF than gastroesophageal reflux disease. The positive impacts of PPI for the treatment of IPF are not supported by well-powered, double-blinded, and multicenter randomized control trials.

## Conclusions

In this systematic review, we aim to highlight three very important aspects of IPF. First, the role of various etiological factors in the development of IPF; second, the role of GERD in the course of IPF; and third, the role of PPIs as a treatment option in IPF irrespective of etiology. In this review, multiple meta-analyses, narrative reviews, and trials such as ASCEND and CAPACITY do support the positive role of PPI on IPF, which serves as a stepping stone for future trials in such field of interest. Additionally, the positive role of GERD is supported by genetics and studies that highlight the fact that the duration for which PPIs are used matters irrespective of etiology. The doctor's responsibility increases whenever the diagnosis of IPF is made. They should make a swift diagnosis, assess for confounding factors and comorbidities, choose a multidisciplinary team for the patient, and provide the best possible care. This systematic review highlights the fact that the importance of PPIs in the treatment of IPF needs to be proved further with well-powered, double-blinded, and multi-center randomized control trials.
